# Occurrence of major earthquakes is as stochastic as smaller ones

**DOI:** 10.1126/sciadv.adx7747

**Published:** 2026-02-11

**Authors:** Zakaria Ghazoui, Jean-Robert Grasso, Arnaud Watlet, Corentin Caudron, Abror Karimov, Yusuke Yokoyama

**Affiliations:** ^1^ British Antarctic Survey, Cambridge, UK.; ^2^ Université Libre de Bruxelles, Brussels, Belgium.; ^3^ ISTerre (Institut des Sciences de la Terre), Université Grenoble Alpes, CNRS, Grenoble, France.; ^4^ Environmental Sensing and Modelling, Luxembourg Institute of Science and Technology, Belvaux, Luxembourg.; ^5^ Atmosphere and Ocean Research Institute, The University of Tokyo, Chiba, Japan.

## Abstract

Seismic hazard estimates rely on interevent time distributions between earthquakes of a given magnitude. In the Himalaya, recurrence intervals are usually modeled as cyclic or quasiperiodic, whereas globally, they range from periodic and clustered to random. Statistical analyses of a 6000-year lake-sediment seismic record, calibrated against regional instrumental data, worldwide paleoseismic records, and synthetic seismic catalogs, demonstrate that time intervals between large earthquakes (*M* ≥ 6.5, based on shaking intensity thresholds calibrated locally) robustly follow a Poisson distribution. Second-order fluctuations indicate event clustering. These observations contradict periodic or quasiperiodic recurrence models. Comparisons with paleoseismic data from other tectonic settings and realistic synthetic catalogs confirm the robustness and broad applicability of these findings. Thus, major earthquakes appear as stochastic as smaller ones, challenging recurrence models derived from limited datasets and substantially increasing seismic hazard estimates.

## Teaser

Paleoseismic and instrumental data converge on Poisson-like recurrence with short-range temporal clustering for large earthquakes.

## INTRODUCTION

Understanding how earthquakes recur is essential for long-term seismic hazard planning, yet two key sources of information tell different stories: Paleoseismic records often point to quasiperiodic recurrence, while instrumental data and statistical physics approaches reveal more complex behaviors, ranging from stochastic to clustered patterns (*1*). Bridging this gap calls for approaches that connect geological archives with physical models to track consistent patterns across contrasting timescales. In instrumental records, this complexity is classically addressed by distinguishing two types of earthquakes: independent (uncorrelated) events and clustered (correlated) sequences triggered by earlier shocks (*1*). These two classes of events are observed at all scales in instrumental seismic catalogs globally. The corresponding interevent time (Δ*t*) distributions fit a power law for short interevent times (i.e., correlated events) and an exponential law for larger values of interevent times, i.e., Poissonian noncorrelated events [for a review, see (*1*)].

**Fig. 1.**
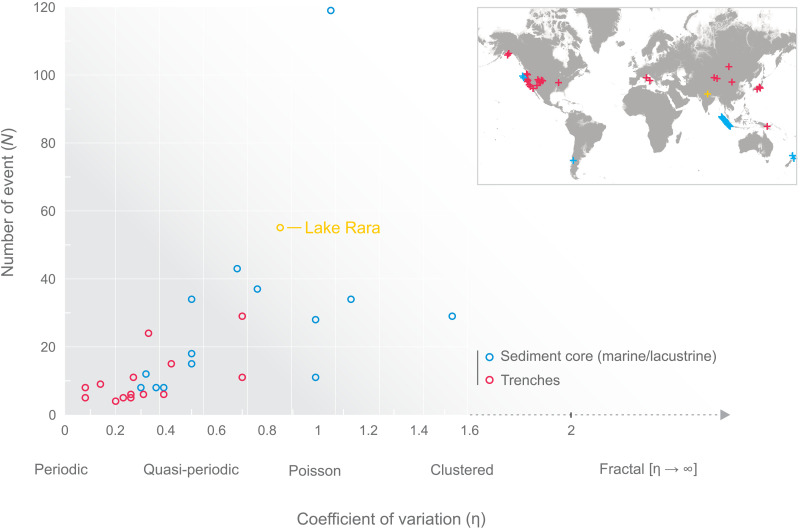
Comparison of the number of events (*N*) versus the coefficient of variation (η) from worldwide paleoseismic records. We compile data from a previous study (*53*) and implemented paleoseismic catalogs from lacustrine to coastal (sediment cores: blue circle and cross; our study site in yellow) via inland sites (trenches; red circle and cross). The different types of seismic behavior defined by the coefficient of variation (η) are indicated below the *x* axis. The coefficient of variation is defined as η = σ/μ, with σ the SD and μ the mean value, e.g., (*31*, *53*, *54*). For Poisson (uncorrelated event) distribution, η = 1; for quasiperiodic to periodic, η < 1; for clustered events (correlated event), η > 1 (*1*).

By contrast, analyses of interevent-time distributions in paleoseismic records worldwide (Fig. 1) have reached divergent conclusions (*2*–*4*). These distributions are thought to encompass a wide range of patterns including (i) quasiperiodic interevent times (*3*, *5*–*7*), (ii) recurrence times varying according to the “supercycle” concept (*8*, *9*), (iii) noncorrelated (Poissonian) distributions (*2*, *10*), and (iv) clustered patterns (*11*). This divergence has likewise been emphasized in recent lacustrine syntheses (*12*, *13*).

With a total length of about 2400 km, the Main Himalayan Thrust and its surface-breaking frontal ramp, the Main Frontal Thrust (MFT), is considered to be the largest and most rapidly slipping continental megathrust worldwide (*14*). Convergence across the Himalayan belt occurs at rates increasing from ~14 to ~21 mm/year from west to east (*15*). The strain accumulated during convergence is released by major earthquakes; the magnitude, time, and location of which remain unpredictable (*14*, *16*). The occurrence of major earthquakes poses a substantial threat to the densely populated Himalayan region and its foreland; therefore, characterizing their return time is both a socioeconomic necessity and a scientific challenge. Most studies have focused on paleoseismic techniques to assess characteristic return times [see (*17*) for a review]. However, along most segments of the MFT, paleoseismic trenches have generally revealed only single events per site on centennial timescales (*17*). In the absence of more complete paleoseismic time series, the mean return times of major earthquakes cannot be robustly ascertained (*17*). In this context and mainly due to lack of evidence or speculative interpretation of insufficient data, the timing of major Himalayan earthquakes remains subject to notable debate (*17*–*23*).

**Fig. 2.**
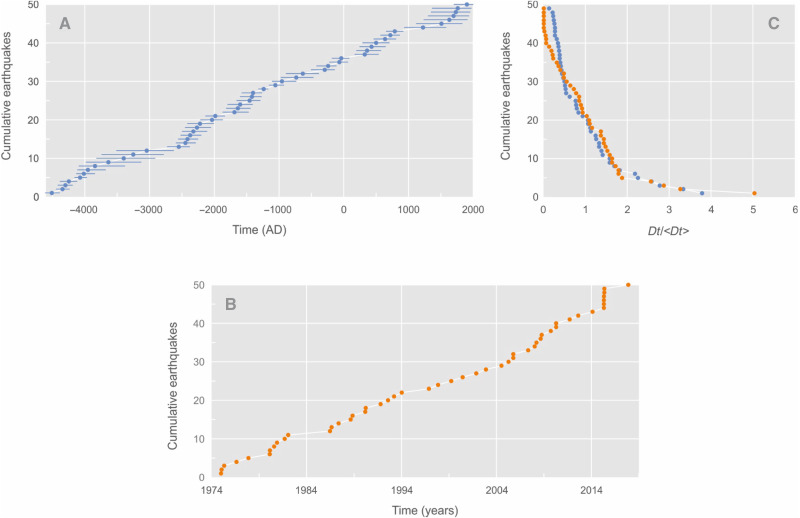
Cumulative number of earthquakes versus time. (**A**) The 50 events of the earthquake-triggered turbidite (ETT) catalog. (**B**) The 50 largest events [moment magnitude (*M*_w_: 6.1 to 7.8] from the US Geological Survey (USGS) instrumental earthquake catalog. (**C**) Collapsed interevent time distributions from both catalogs obtained by rescaling the interevent times by the mean interevent time of each distribution (orange dots: USGS instrumental earthquake catalog; blue dots: ETT catalog). Events are from the area defined in Fig. 1.

Here, we use statistical physics and statistical seismology to (i) characterize the temporal distribution of 50 seismic events recorded in a 6000-year lacustrine sediment record from Lake Rara (western Nepal) and (ii) test the robustness and generality of these results. For the latter, we compare them with paleoseismic records from other active tectonic regions and with synthetic earthquake catalogs [Fig. 2A; and figs. S1 to S3; see also (*24*)].

## RESULTS AND DISCUSSION

The paleoseismic catalog is built on a 4-m-long sediment core retrieved from Lake Rara in western Nepal (figs. S1 and S3). Radiocarbon dating shows that the long sediment core covers 6000 years of sedimentary history (*24*) and is punctuated by a series of 50 turbidites (Fig. 2A and figs. S2 and S3). Turbidites were identified on the basis of geochemical profiles obtained by x-ray fluorescence (XRF), visual inspection, and enhanced photographs of the core [fig. S3; see the Supplementary Materials and (*25*)]. We consider that all the major turbidites have been identified, although it is likely that some turbidites with smaller thickness and/or occurring in time very close to another remain undetected. The 2σ (95% confidence level) uncertainty on timing related to the age-depth model is presented in the Supplementary Materials (fig. S4), and the numerical 2σ age ranges of every event are also listed in table S1. On the basis of the same age-depth model, we also present the minimum and maximum ages for each of the 50 turbidites (Fig. 2A and table S1). The 50 turbidites within the sediment core are inferred to have been triggered by regional earthquakes (*25*). The main arguments supporting this interpretation are the observed synchronicity with large modern regional earthquakes and the low local susceptibility to floods and landslides (*25*). Ghazoui *et al.* (*25*) constrained the local shaking-intensity threshold to trigger a turbidite within Lake Rara slope to Modified Mercalli Intensity (MMI) > 5.5 ± 0.2, based on observed and modeled intensity maps. This threshold corresponds to near-field earthquakes with a *M*_w_ ≥ 5.6 or regional earthquakes with *M*_w_ ≥ ~6.5 within a 150- to 200-km distance range (Fig. 1). For concision, the resulting 50-event earthquake-triggered turbidite (ETT) catalog is hereafter referred to as the ETT catalog.

To calibrate the ETT time series (Fig. 2A) against instrumental seismic catalogs (Fig. 2B) for which event size is known, we use the US Geological Survey (USGS) regional seismicity catalog (https://earthquake.usgs.gov/earthquakes/search/). We selected subsets of earthquakes in three distinct zones to generate regional catalogs. Two of these zones are circular areas centered on Lake Rara (29.53°N and 82.09°E), with radii of 200 and 500 km, respectively, chosen to capture local and broader seismicity around the central Himalaya (figs. S2 and S5). The third zone is a rectangular box (26.68°N to 37.03°N and 72.87°E to 97.23°E) that encompasses the full Himalayan arc and most of southern Tibet (Fig. 2B and fig. S2), providing a regional-scale comparison. To avoid magnitude completeness issues for the catalogs (*26*, *27*) and to allow a quantitative comparison with the ETT catalog, we selected the 50 largest instrumental events for each subcatalog. To quantitatively compare the two distributions (Fig. 2C and fig. S5), we rescale the interevent times by the mean interevent time of each distribution (*11*). The collapse of the normalized interevent time distributions we observe (Fig. 2C and fig. S5) supports the scaling behavior between a regional (2300 km by 1300 km, 44 years) instrumental series (Fig. 2C and figs. S2 and S5) and the local (~200-km radius; fig. S5) catalogs for both 44 years and the 6000-year time window. These scaling collapses support (i) the classical ergodicity assumption for seismicity (*28*, *29*) and (ii) the scaling properties of earthquake dynamics (*11*). For the instrumental catalogs, the magnitude bandwidth for each of the three seismic zones is defined as Δ*M* = *M*_max_ − *M*_c_, where *M*_max_ is the maximum magnitude in the catalog and *M*_c_ is the threshold magnitude value for recording completeness. Across all three seismic zones, this bandwidth falls within the 1.9 ± 0.5 range (see the Calibration section in Materials and Methods). This pattern is observed when the time series correspond to catalogs with a small number of aftershocks, the results from using a narrow magnitude bandwidth (Δ*M* ≤ 2) for event recording (*11*, *30*). While it is not possible to relate each of the ETT events to a given magnitude, we alternatively estimate the Δ*M* value for the whole ETT catalog by analogy with the instrumental datasets.

The primary way to characterize the interevent time distribution is based on the coefficient of variation [η = σ/μ, with σ the SD and μ the mean value, e.g., (*31*, *32*)]. Kagan and Jackson (*33*) demonstrate a Poisson (uncorrelated events) distribution as having η = 1, whereas distributions with η < 1 are quasiperiodic to periodic, and those with η > 1 correspond to clustered events (correlated events). For the 50 ETT events, this ratio is close to unity (η = 0.90 ± 0.091; Fig. 1), indicating a uniform distribution of event times. To the first order, the η = 0.90 ± 0.091 value rules out periodic event patterns for the ETT events (*11*, *33*, *34*). When interpreted in terms of instrumental earthquake time series, this implies that the magnitude range of recorded earthquakes is too narrow to capture triggered earthquake clustering (*30*, *35*, *36*).

**Fig. 3.**
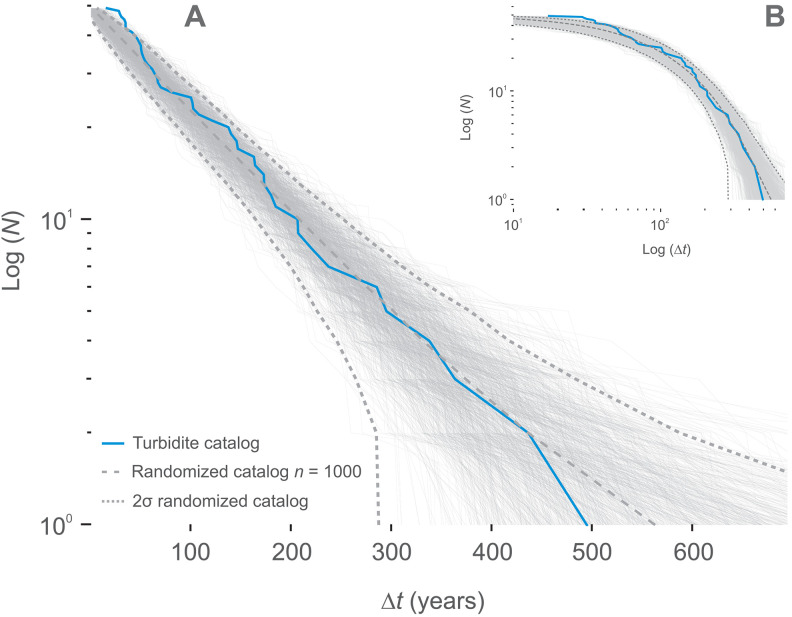
Plots of the cumulative distributions of earthquakes (*N*) as a function of interevent time (Δ*t*). (**A**) Log-linear and (**B**) log-log plot for the ETT catalog. Exponential distributions reproduce the data for both the ETT catalog (A and B; blue curve). Gray bold lines are distributions from randomized (synthetic) catalog (*n* series of 50 event sequence; *n* = 1000) bracket between original *t*_min_ and *t*_max_. The dark gray dashed curves represent the 2σ (95%) confidence level of the exponential fit. The central dashed gray curve represents the absolute reference to the Poisson model. Note the presence of expected border effect exhibiting the resolution limit for very short time intervals (*39*). The instrumental data are shown on fig. S3 (C and D).

**Fig. 4.**
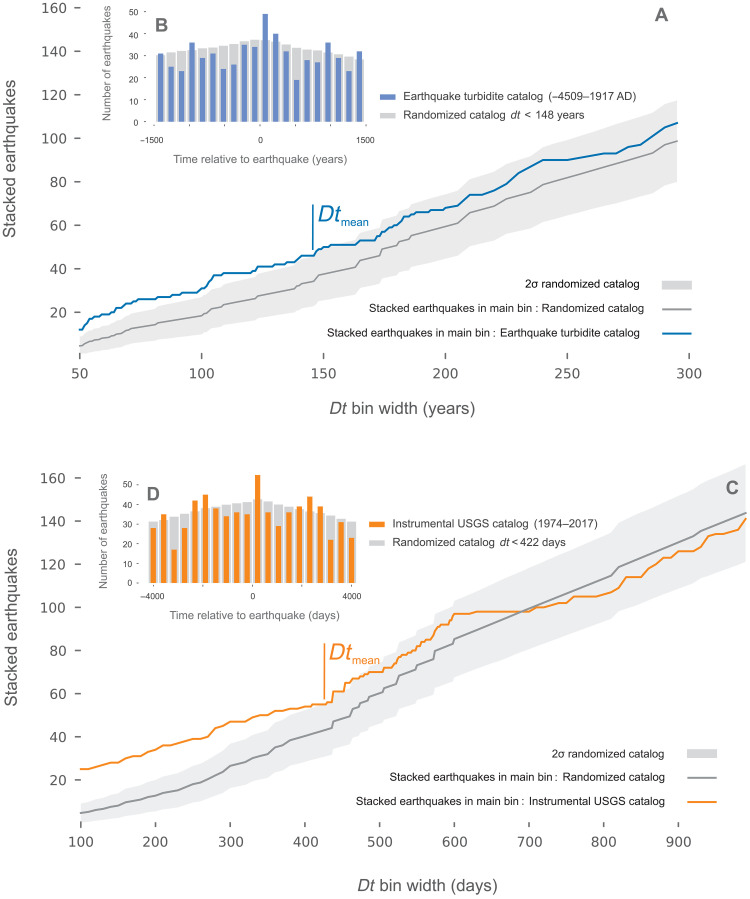
Quantification of earthquake cluster triggering in both catalogs. Stacked time series for (**A** and **B**) ETT and (**C** and **D**) instrumental (USGS) catalogs. (A and C) A cluster is characterized in time by (A) the stacked ETT (blue line) and (C) the stacked instrumental (orange line) events that emerge from the 2σ (95%) randomized (synthetic) catalogs (*n* = 1000 events; gray envelope). The mean duration *Dt*_mean_ for (A) the earthquake turbidite cluster corresponds to 148 years and (C) for the instrumental earthquake USGS catalog is 422 days. We define a cluster with a minimum of two events separated by less than *Dt*_mean_ value for both catalogs. For the stack (B) and (D), we assign a common *t*_0_ value to each reference event of the series, and we sum the series before and after *t*_0_. The gray histogram represented respectively the randomized (synthetic) catalog for (A) to (D). The gray envelope represents the 95% confidence range; the first point where the colored curve reenters this envelope defines the mean cluster duration *Dt*_mean_, which is subsequently used as the threshold for assigning events to the same cluster (tables S2 and S3).

These patterns are confirmed by testing the interevent time distribution for the ETT events against randomized (synthetic) distributions (Fig. 3 and fig. S6; see the Generation of reference (null) catalogs by randomization section in Materials and Methods). A purely Poisson process yields an exponential interevent-time distribution; on a log-linear cumulative plot, this appears as a straight line. We therefore compare the ETT empirical curves with the Poisson line to detect any systematic deviation. The observed series, for both the instrumental and the ETT catalogs, are consistent with a Poisson distribution at 2σ (95%) confidence level, as represented by the theoretical curve of the Poisson model (Fig. 3A and fig. S6C). The Poisson distribution of the corresponding earthquake times indicates that these noncorrelated events are primarily driven by an external driving force (i.e., the tectonic plate deformations), with a weak contribution of interactions with others events (i.e., triggering by other earthquakes) (*11*, *30*). The slight upward curvature at *dt* < *Dt*_mean_ visible in both datasets (Fig. 3, A and C) is the signature of a correlated component in the interevent-time distribution (later isolated and quantified as seismic clusters; Fig. 4). Accordingly, deviations from the Poisson-process expectation (Fig. 3A and fig. S6C) correspond to the correlated events within the series. These patterns are identified in both the ETT and the instrumental catalogs.

To quantify the contribution of short, correlated interevent times (i.e., temporal clustering) in our earthquake series, we applied a superposed epoch analysis, also known as event stacking [Fig. 4 and (*11*)]. For this approach, each earthquake in the series is treated, in turn, as a “reference event” (or “trigger event”), and the occurrence times of all other earthquakes are measured relative to it within a symmetric window of size ±Δ*t*. At each time offset from the reference event, we count the number of subsequent earthquakes (“event counts”) and then average these counts across all reference events, producing a cumulative curve that defines the average pre- and postevent recurrence patterns, respectively. To assess the statistical significance of these patterns, we used the same technique to construct a 2σ (95%) confidence envelope using 1000 synthetic (randomized Poisson) catalogs, computed by randomly redistributing all events while preserving catalog duration and event count. Any portion of the observed cumulative curve that lies above this envelope indicates statistically significant clustering beyond random expectations. The duration of statistically significant clustering (“mean cluster duration”) is defined as the first time point after the reference event when the cumulative curve returns below the 2σ (95%) confidence envelope. In our data, the postevent rate remains elevated from *t* = 0 to approximately Δ*t* ≈ 300 years, with the first full return to the confidence envelope at *Dt*_mean_ = 148 years for the ETT catalog and at 422 days for the instrumental catalog (tables S2 and S3; Fig. 4A and fig. S6A). Within these intervals, the postevent rate remains significantly elevated, indicating temporally clustered recurrence that cannot be explained by a memoryless (Poisson) process (see the Time distribution analysis section in Materials and Methods). The same patterns are resolved using the USGS catalog in the 100- to 400-day window. These patterns are robust at the 2σ (95%) confidence level against randomized series (Fig. 3, A and C). Accordingly, this ETT catalog is equivalent in time to a seismic catalog with dampened or filtered aftershocks. The analyses of time patterns strongly suggest the ETT catalog, and its related Poisson interevent time distribution, to be a proxy for a regional earthquake catalog in which only the largest events are recorded.

A first-order calibration of Δ*M* can be derived from the overlap between the normalized *dt* distribution patterns we resolve for the three instrumental and ETT catalogs (Fig. 2C and fig. S5). Specifically, we (i) normalized each catalog by its mean interevent time, (ii) superposed the cumulative *dt*/⟨*dt*⟩ curves (Fig. 2C and fig. S5), and (iii) retained the instrumental subset whose curve shows the smallest integral vertical deviation from the ETT curve. That subset is the *R* = 200-km catalog, characterized by Δ*M* = 1.9 ± 0.2; adopting the full 1.6 to 2.5 spread of all three instrumental subsets yields Δ*M* ≈ 1.9 ± 0.5 for the ETT catalog.

It is well established that, in a seismic catalog, the number of correlated earthquakes (such as aftershocks) increases as the magnitude bandwidth of the catalog (Δ*M*) becomes larger (*11*). The collapse of the *dt* distributions we resolve supports that the turbidite dataset overlaps with earthquake time series for which Δ*M* is in the 1.5-to-2.4 range, i.e., Δ*M* ≈ 1.9 ± 0.5. By normalizing each catalog by its mean interevent time and overlaying the cumulative-distribution curves of the resulting *dt*/⟨*dt*⟩ values (Fig. 2C and fig. S5), we observe that the curve of the *R* = 200-km instrumental subset is indistinguishable from the ETT curve at plotting resolution, while the wider instrumental catalog subsets plot systematically above or below. This overlap indicates that the two datasets contain the same fraction of correlated events; hence, we assign to the ETT catalog the Δ*M* of the 200-km subset (1.9 ± 0.2), and we retain a ±0.5 margin to span the full 1.6-to-2.5 range covered by all three subsets. Using a lower-bound estimate for ETT *M*_max_ = 8.2 [*M* in the 8.2-to-8.4 range for 1505 western Nepal earthquake; e.g., (*37*–*40*)], the recording threshold for the ETT catalog completeness would thus be in the range of *M*_c_ = *M*_max_ – 1.9, i.e., *M*_w_: 6.3 to 6.8; this value is consistent with earlier estimates based on modeling shaking intensities of known earthquakes at the lake (*25*).

Within the overall random (Poisson-like) distribution observed in the ETT catalog, we applied superposed epoch analysis to systematically identify significant event clustering. This analysis revealed that, in the 6000-year-long ETT catalog, the earthquake occurrence rate remains significantly elevated above the randomized (null) series for intervals up to 150 years after each trigger event (see Fig. 4 and table S2). A similar trend for clustering pattern is observed in the 44-year Himalaya USGS instrumental catalog, where the postevent rate remains above the random expectation for intervals up to 420 days following a mainshock (see Fig. 4 and fig. S6).

Using the 150-year correlation window allows us to characterize the ETT catalog as a succession of 33 correlated and 17 uncorrelated events (Fig. 4A, fig. S7A, and tables S2 and S3). Similar results are obtained for the instrumental catalog (32 correlated and 18 uncorrelated events). Using the *Dt*_mean_ = 148-year criterion (Fig. 4), the 6000-year catalog breaks into 12 clusters whose onsets are separated by 11 intercluster intervals ranging from 173 to 722 years (mean = 395 ± 180 years; table S2). These values are descriptive summaries of the data under the assumption that events separated by less than *Dt*_mean_ apart belong to the same cluster and should not be interpreted as fixed recurrence periods. The largest (1200 year) interevent time highlights how the wide range of possible interevent time values may bias recurrence interval estimates when the number of data is small.

**Fig. 5.**
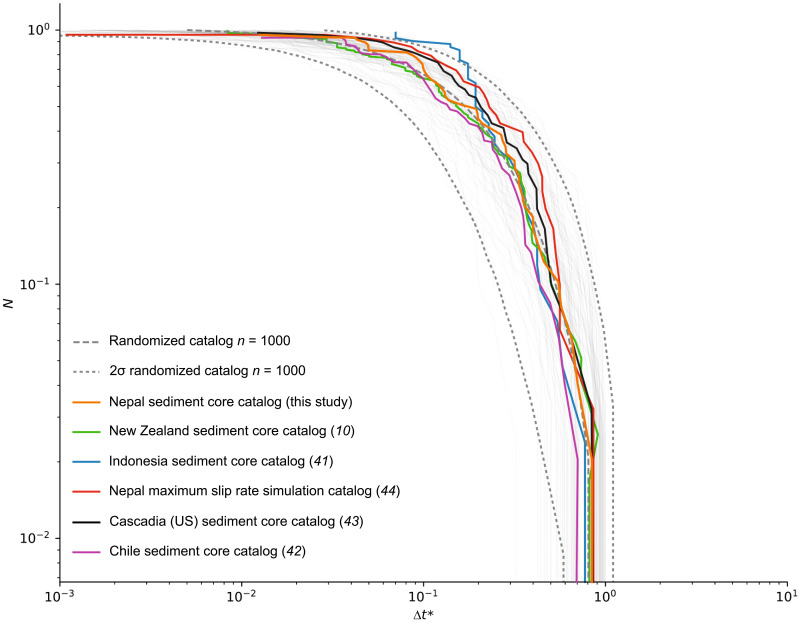
Comparison of the cumulative distributions of earthquakes (*N*) as a function of normalized interevent time (Δt*) from worldwide paleoseismic and synthetic catalog. We compile interevent time distributions from the paleoseismic catalogs with the largest numbers of earthquakes (*N*). These include lake and marine sediment core records from Nepal (this study), New Zealand (*10*), Indonesia (*41*), Chile (*42*), and Cascadia, USA (*43*). We also incorporate a synthetic catalog based on the rupture model of the 2015 Nepal Gorkha earthquake (*44*). At this, we compared them to the interevent time distribution obtained from a simple rupture model developed for the Gorkha earthquake. Log-log plot for paleoseismic and synthetic catalogs. Exponential distributions reproduce the data for both the ETT catalog (blue curve). Gray bold lines are distributions from randomized (synthetic) catalogs (*n* series of event sequence; *n* = 1000) bracket between original *t*_min_ and *t*_max_. The dark gray dashed curves represent the 2σ (95%) confidence level of the exponential fit. The central dashed gray curve represents the absolute reference to the Poisson model. Note the presence of expected border effect exhibiting the resolution limit for very short time intervals (*39*).

When we compare our overall Poissonian distribution with global paleoseismic time series (Fig. 1), we observe a significant correlation [correlation coefficient (*r*) = 0.591 and *P* < 0.0007] between the number of recorded events and η. Thus, the larger the number of recorded events in a series, the more uniform the observed time distribution (i.e., no periodicity). When the number of events in a dataset increases (Fig. 1), the observed time pattern moves from a periodic or quasiperiodic organization (η < 1) to Poisson-like (η = 1) and subsequently clustered (η > 1) patterns. These results question the robustness of analyses based on small datasets size. The comparison of paleoseismic datasets from New Zealand (*10*), Indonesia (*41*), Chile (*42*), and Cascadia [USA (*43*)], as shown in Fig. 5, confirms the robustness of both the Poissonian distribution and short-range clustering seen at Lake Rara. These statistical patterns—a general Poisson distribution with clustered recurrence at short timescales—are consistently found in all examined long turbidite earthquake catalogs, irrespective of the tectonic setting. A synthetic catalog based on the 2015 Gorkha earthquake rupture model (*44*) further illustrates that earthquake sequences generated under a realistic rupture scenario align with the statistical patterns identified in paleoseismic records (Fig. 5). The synthetic catalog generated from the rupture model produces interevent-time distributions that overlap with those of our paleoseismic records, suggesting that the statistical features we observe reflect genuine earthquake sequence dynamics rather than artifacts of local record incompleteness. This provides additional support for interpreting the Lake Rara turbidite series as representative of earthquake-triggered sequences.

This interpretation aligns well with existing global statistical models of seismicity, including unified scaling and power-law distributions previously proposed by Bak *et al.* (*45*), Corral and González (*46*), and Navas-Portella *et al.* (*47*) and thereby reinforcing the scientific validity of our statistical approach.

Both the 6000-year ETT and the 44-year instrumental time distributions are dominated by Poissonian processes, with fluctuations corresponding to clusters of correlated events. This result challenges the model of cycles and quasiperiodicity that is suggested by numerous paleoseismological studies. In terms of seismic hazard, our results imply that the occurrence of major earthquakes is as uncertain (Poissonian) as the one of moderate to small earthquakes, irrespective of the time scale considered, thereby considerably increasing seismic hazard and exposing Himalayan countries to a permanent threat of large to major earthquakes.

On the global scale, this study bridges the gap between analyses based on instrumental seismic catalogs (large numbers of recent earthquakes), synthetic catalog, and catalogs based on paleoseismic data (small number of historical earthquakes on large time scales). By explicitly addressing previous methodological criticisms related to data completeness of paleoseismic records (*12*, *13*), we validate the broader relevance of instrumental statistical seismology approaches for analyzing paleoseismic records. This highlights potential biases inherent in small paleoseismic datasets and confirms the necessity of applying statistically robust methods from instrumental seismology to paleoseismic data. Ultimately, these findings underscore the inherent complexity required in any seismic cycle conceptual model, with important implications for seismic risk assessment and hazard mitigation strategies. In doing so, we explicitly address earlier concerns regarding paleoseismic data completeness and catalog length that limit the reliability of recurrence analyses [see, e.g., (*12*, *13*)]. By combining a large-*N*, long-term catalog with randomized statistical testing to quantify uncertainties, we validate the broader relevance of instrumental seismology methods applied to paleoseismic data. Our results derived from paleoseismic data align closely instrumental patterns, emphasizing the inherent complexity and stochastic nature of earthquake recurrence, beyond the time windows captured by instrumental earthquake catalog.

## MATERIALS AND METHODS

### ETT catalog construction

#### Identification of turbidite layers

The turbidites were identified by Nakamura *et al*. (*24*), and the recognition was improved on the basis of the multiproxy study of Ghazoui *et al.* (*25*), which used a series of sedimentological and geochemical criteria that included grain size, magnetic susceptibility, Ti concentrations, bulk organic geochemistry, and radio density to identify turbidites in short cores from the same lake. The turbidites were identified by their coarse base and a fining-upward sequence of fine sand to very fine silt in sharp contact with the underlying background mud. It has been shown that Ti concentrations reflect silt content in sediments, (*40*, *48*) and the Ti/Ca ratio proved to be a reliable turbidite indicator in our previous study (*25*). We therefore use the Ti/Ca ratio as a turbidite proxy in the long core also. We calibrated these measurements with the descriptive log (*24*) and an enhanced core photograph (fig. S2). The enhancement consists of calibrating the value of the white balance on the bright mica layer, which highlights the turbidite layers.

#### Age model

A chronology for the Lake Rara long sediment record was established on core RA09-04 by radiocarbon dating [see (*24*) for the complete procedure]. Samples for radiocarbon dating were picked outside of the turbidites, as these are considered to be instantaneous deposits (*24*). All ages were calibrated using the calibration curve for Northern Hemisphere terrestrial ^14^C dates IntCal13 (*49*). We updated the age-depth model of core RA09-04 (fig. S2) using a Bayesian model (*50*) after removal of the turbidites, which allows taking prior assumptions on accumulation rate and its variability through time into account and provides formal uncertainties on the turbidite ages (*50*).

### Time distribution analysis

We aim to extract patterns that characterize the past 6000 years of seismicity around Lake Rara (western Nepal) from the ETT catalog. We analyze the interevent time distribution to quantify the level of event interactions, i.e., whether the interevent time distribution shows a clustered, periodic, or Poissonian pattern (Fig. 3). As emphasized by de Arcangelis *et al.* (*1*), the functional form and scaling of interevent (Δ*t*) distributions are fundamental signatures of underlying physical processes that control seismicity, including triggering, cluster effects, and external driving. Because the catalog contains a relatively limited number of events, we use a suite of complementary statistical techniques, including the coefficient of variation, superposed epoch stacking, and comparison with ensemble-based null models to constrain the inferred event interaction regime and increase the signal-to-noise ratio of the catalog. For this purpose, we stack the time series (fig. S4) in a superposed epoch analysis (*11*, *51*, *52*) to resolve any possible clustering in the ETT catalog (Fig. 4 and fig. S4). Last, we compare the interevent time patterns resolved using our ETT catalog to the distribution reported from other analyses of worldwide paleoseismicity time series using the coefficient of variability (i.e., SD/mean).

#### Calibration

Using the USGS instrumental database and the statistical laws that drive earthquake interactions (i.e., Gutenberg-Richter, Omori’s and Bath’s laws), we aim to bound the Δ*M* value, i.e., the difference between the maximum recorded magnitude *M*_max_ and the threshold magnitude for recording completeness *M*c (ΔM = *M*_max_ – *M*_c_), which emerges from the turbidite time series. This controlled calibration ensures that comparisons between paleo- and instrumental catalogs reflect true variations in temporal organization rather than artifacts of magnitude completeness or event detection thresholds (*1*). Instrumental catalogs show that the proportion of aftershocks increases sharply when the magnitude bandwidth Δ*M* of the catalog reaches or exceeds approximately two magnitude units. In this case, both mainshocks and their largest aftershocks are recorded and cataloged. In contrast, when the bandwidth is narrower (Δ*M* ≤ 1.5 to 2), many aftershock magnitudes fall below the catalog’s completeness threshold and are thus not recorded. According to Bath’s law, the largest aftershock in a sequence is on average about 1.2 ± 0.3 magnitude units smaller than its mainshock (*1*).

#### Generation of reference (null) catalogs by randomization

To assess the statistical significance of observed temporal patterns in earthquake occurrence, ensembles of 1000 reference (null) catalogs were generated for both the paleoseismic (ETT) and instrumental (USGS) catalogs. Each synthetic catalog replicated the observed number of events and maintained the exact temporal extent of the original dataset. For every synthetic catalog, each event time was independently drawn from a continuous uniform distribution, spanning the entire observational window as defined by the real catalog. This protocol ensures that all temporal correlations, memory effects, or clustering are systematically eliminated while maintaining the empirical event density and boundary constraints.

This approach builds fully synthetic series where each event time is generated independently, thereby instituting a strict null hypothesis reflecting a homogeneous, Poissonian (memoryless) process. This methodological choice is directly aligned with statistical seismology best practice and the framework advanced by de Arcangelis *et al.* (*1*), who recommend such fully randomized catalogs as optimal null models for testing the significance of clustering or periodicity.

For each catalog, the same statistical analyses as applied to the real series—such as interevent time distributions and superposed epoch (stacking) analyses—were conducted. Summary statistics from the ensemble of null catalogs were used to construct empirical confidence envelopes [e.g., 2σ (95%), calculated as the 2.5th to 97.5th percentiles at each interval]. Statistically significant deviations in the observations were defined by excursions outside these envelopes, marking the presence of clustering or temporal organization exceeding the expectation from a purely random process.

A central motivation for using the randomization protocol arises from concerns over the propagation of age-model uncertainties in paleoseismic data, an issue highlighted by Kempf and Moernaut (*13*). By emphasizing ensemble-level statistical behavior (e.g., the overall shape of interevent time distributions or stacking curves) rather than the precise timing of individual events, our approach considerably reduces the influence of individual age uncertainties on the main interpretations. These collective indicators are robust so long as age uncertainties are moderate and do not systematically bias the series on the analyzed timescales. This methodological choice is validated both by recent statistical seismology literature and by empirical tests, ensuring that our detection of significant departures from Poissonian (random) sequences genuinely reflects temporal organization in the data and not artifacts of chronological imprecision (*12*).

#### Superposed-epoch analysis and cluster definition

To quantify the contribution of correlated events, the superposed-epoch (i.e., temporal clustering) analysis consists of the following steps: (i) Take each earthquake in the catalog, in turn, as a reference event; (ii) compute the relative occurrence times of all other earthquakes with respect to the reference event; and (iii) bin these time differences into symmetric, fixed-width intervals spanning ±Δ*t*, and count the number of events falling within each bin.

The binned counts are then averaged over all reference events to produce the cumulative curves shown in Fig. 4 (A and C). Then, the cluster significance assessment is performed by repeating the procedure on 1000 randomized synthetic catalogs (redistributing events randomly within the observation window while preserving catalog duration and count). A 2σ (95%) confidence envelope is built from the randomized catalogs. The first intersection between the observed curve and the envelope defines *Dt*_mean_, the mean cluster duration. Two successive events are assigned to the same cluster if their interevent time is <*Dt*_mean_. The resulting cluster and intercluster statistics are summarized in tables S2 and S3. All statistics that involve intercluster times (e.g., the 173- to 722-year range reported in Results and Discussion) therefore remain contingent on the chosen value of *Dt*_mean_; selecting a different *Dt*_mean_ would proportionally shift the interval range.

## Supplementary Materials

### This PDF file includes:

Supplementary Text Figs. S1 to S7 Tables S1 to S3 References

## References

[1] L. de Arcangelis, C. Godano, J. R. Grasso, E. Lippiello, Statistical physics approach to earthquake occurrence and forecasting. Phys. Rep. 628, 1–91 (2016).

[2] S. C. Wu, C. A. Cornell, S. R. Winterstein, A hybrid recurrence model and its implication on seismic hazard results. Bull. Seismol. Soc. Am. 85, 1–16 (1995).

[3] L. R. Sykes, W. Menke, Repeat times of large earthquakes: Implications for earthquake mechanics and long-term prediction. Bull. Seismol. Soc. Am. 96, 1569–1596 (2006).

[4] K. Satake, B. F. Atwater, Long-term perspectives on giant earthquakes and tsunamis at subduction zones. Annu. Rev. Earth Planet. Sci. 35, 349–374 (2007).

[5] K. M. Scharer, G. P. Biasi, R. J. Weldon, T. E. Fumal, Quasi-periodic recurrence of large earthquakes on the southern San Andreas fault. Geology 38, 555–558 (2010).

[6] K. R. Berryman, U. A. Cochran, K. J. Clark, G. P. Biasi, R. M. Langridge, P. Villamor, Major earthquakes occur regularly on an isolated plate boundary fault. Science 336, 1690–1693 (2012). 22745426 10.1126/science.1218959

[7] F. Corbi, F. Funiciello, M. Moroni, Y. van Dinther, P. M. Mai, L. A. Dalguer, C. Faccenna, The seismic cycle at subduction thrusts: 1. Insights from laboratory models. J. Geophys. Res. 118, 1483–1501 (2013).

[8] C. Goldfinger, Y. Ikeda, R. S. Yeats, J. J. Ren, Superquakes and supercycles. Seismol. Res. Lett. 84, 24–32 (2013).

[9] R. Herrendorfer, Y. van Dinther, T. Gerya, L. A. Dalguer, Earthquake supercycle in subduction zones controlled by the width of the seismogenic zone. Nat. Geosci. 8, 471–473 (2015).

[10] B. Gomez, Á. Corral, A. R. Orpin, M. J. Page, H. Pouderoux, P. Upton, Lake Tutira paleoseismic record confirms random, moderate to major and/or great Hawke’s Bay (New Zealand) earthquakes. Geology 43, 103–106 (2015).

[11] S. J. Kenner, M. Simons, Temporal clustering of major earthquakes along individual faults due to post-seismic reloading. Geophys. J. Int. 160, 179–194 (2005).

[12] J. Moernaut, Time-dependent recurrence of strong earthquake shaking near plate boundaries: A lake sediment perspective. Earth Sci. Rev. 210, 103348 (2020).

[13] P. Kempf, J. Moernaut, Age uncertainty in recurrence analysis of Paleoseismic records. J. Geophys. Res. Solid Earth 126, e2020JB021774 (2021).

[14] R. Cattin, J. P. Avouac, Modeling mountain building and the seismic cycle in the Himalaya of Nepal. J. Geophys. Res. 105, 13389–13407 (2000).

[15] V. L. Stevens, J. P. Avouac, Interseismic coupling on the Main Himalayan Thrust. Geophys. Res. Lett. 42, 5828–5837 (2015).

[16] R. Bilham, V. K. Gaur, P. Molnar, Himalayan seismic hazard. Science 293, 1442–1444 (2001). 11520972 10.1126/science.1062584

[17] L. Bollinger, S. N. Sapkota, P. Tapponnier, Y. Klinger, M. Rizza, J. Van der Woerd, D. R. Tiwari, R. Pandey, A. Bitri, S. Bes de Berc, Estimating the return times of great Himalayan earthquakes in eastern Nepal: Evidence from the Patu and Bardibas strands of the Main Frontal Thrust. J. Geophys. Res. 119, 7123–7163 (2014).

[18] T. Ader, J.-P. Avouac, J. Liu-Zeng, H. Lyon-Caen, L. Bollinger, J. Galetzka, J. Genrich, M. Thomas, K. Chanard, S. N. Sapkota, S. Rajaure, P. Shrestha, L. Ding, M. Flouzat, Convergence rate across the Nepal Himalaya and interseismic coupling on the Main Himalayan Thrust: Implications for seismic hazard. J. Geophys. Res. 117, B04403 (2012).

[19] Y. Kumahara, R. Jayangondaperumal, Paleoseismic evidence of a surface rupture along the northwestern Himalayan Frontal Thrust (HFT). Geomorphology 180, 47–56 (2013).

[20] J.-L. Mugnier, A. Gajurel, P. Huyghe, R. Jayangondaperumal, F. Jouanne, B. Upreti, Structural interpretation of the great earthquakes of the last millennium in the central Himalaya. Earth Sci. Rev. 127, 30–47 (2013).

[21] H. N. Srivastava, B. K. Bansal, M. Verma, Largest earthquake in Himalaya: An appraisal. J. Geol. Soc. India 82, 15–22 (2013).

[22] C. Schiffman, B. S. Bali, W. Szeliga, R. Bilham, Seismic slip deficit in the Kashmir Himalaya from GPS observations. Geophys. Res. Lett. 40, 5642–5645 (2013).

[23] S. G. Wesnousky, Y. Kumahara, D. Chamlagain, I. K. Pierce, T. Reedy, S. J. Angster, B. Giri, Large paleoearthquake timing and displacement near Damak in eastern Nepal on the Himalayan Frontal Thrust. Geophys. Res. Lett. 44, 8219–8226 (2017).

[24] A. Nakamura, Y. Yokoyama, H. Maemoku, H. Yagi, M. Okamura, H. Matsuoka, N. Miyake, T. Osada, H. Teramura, D. P. Adhikari, V. Dangol, Y. Miyairi, S. Obrochta, H. Matsuzaki, Late Holocene Asian monsoon variations recorded in Lake Rara sediment, western Nepal. J. Quat. Sci. 27, 125–128 (2012).

[25] Z. Ghazoui, S. Bertrand, K. Vanneste, Y. Yokoyama, J. Nomade, A. P. Gajurel, P. A. van der Beek, Potentially large post-1505 AD earthquakes in western Nepal revealed by a lake sediment record. Nat. Commun. 10, 2258 (2019). 31113962 10.1038/s41467-019-10093-4 PMC6529449

[26] Y. Y. Kagan, P. Bird, D. D. Jackson, Earthquake patterns in diverse tectonic zones of the globe. Pure Appl. Geophys. 167, 721–741 (2010).

[27] M. Tahir, J. R. Grasso, Aftershock patterns of *M*_s_>7 earthquakes in the India-Asia collision belt: Anomalous results from the Muzaffarabad earthquake sequence, Kashmir, 2005. Bull. Seismol. Soc. Am. 104, 1–23 (2014).

[28] I. Main, Statistical physics, seismogenesis, and seismic hazard. Rev. Geophys. 34, 433–462 (1996).

[29] J. G. Anderson, J. N. Brune, Methodology for using precarious rocks in Nevada to test seismic hazard models. Bull. Seismol. Soc. Am. 89, 456–467 (1999).

[30] A. Helmstetter, Is earthquake triggering driven by small earthquakes? Phys. Rev. Lett. 91, 058501 (2003). 12906641 10.1103/PhysRevLett.91.058501

[31] T. F. Cox, T. Lewis, Conditioned distance ratio method for analyzing spatial patterns. Biometrika 63, 483–491 (1976).

[32] W. Marzocchi, L. Zaccarelli, A quantitative model for the time-size distribution of eruptions. J. Geophys. Res. 111, B04204 (2006).

[33] Y. Y. Kagan, D. D. Jackson, Long-term earthquake clustering. Geophys. J. Int. 104, 117–133 (1991).

[34] J. K. Gardner, L. Knopoff, Sequence of earthquakes in southern California, with aftershocks removed, Poissonian. Bull. Seismol. Soc. Am. 64, 1363–1377 (1974).

[35] P. Traversa, J. R. Grasso, Brittle creep damage as the seismic signature of dyke propagations within basaltic volcanoes. Bull. Seismol. Soc. Am. 99, 2035–2043 (2009).

[36] M. Tahir, J. R. Grasso, D. Amorese, The largest aftershock: How strong, how far away, how delayed? Geophys. Res. Lett. 39, L20308 (2012).

[37] N. Ambraseys, D. Jackson, A note on early earthquakes in northern India and southern Tibet. Curr. Sci. 84, 570–582 (2003).

[38] R. Bilham, K. Wallace, Future Mw>8 earthquakes in the Himalaya: Implications from the 26 Dec 2004 Mw=9.0 earthquake on India’s eastern plate margin. Geol. Surv. India Spec. Publ. 85, 1–14 (2005).

[39] S. Kumar, S. G. Wesnousky, R. Jayangondaperumal, T. Nakata, Y. Kumahara, V. Singh, Paleoseismological evidence of surface faulting along the northeastern Himalayan front, India: Timing, size, and spatial extent of great earthquakes. J. Geophys. Res. 115, B12422 (2010).

[40] V. L. Stevens, J. P. Avouac, Millenary *M_w_*>9.0 earthquakes required by geodetic strain in the Himalaya. Geophys. Res. Lett. 43, 1118–1123 (2016).

[41] J. R. Patton, C. Goldfinger, A. E. Morey, K. Ikehara, C. Romsos, J. Stoner, Y. Djadjadihardja, Udrekh, A. Sri, E. Z. Gaffar, A. Vizcaino, A 6600 year earthquake history in the region of the 2004 Sumatra-Andaman subduction zone earthquake. Geosphere 11, 2067–2129 (2015).

[42] J. Moernaut, M. Van Daele, K. Fontijn, K. Heirman, P. Kempf, M. Pino, G. Valdebenito, R. Urrutia, M. Strasser, M. De Batist, Larger earthquakes recur more periodically: New insights in the megathrust earthquake cycle from lacustrine turbidite records in south-central Chile. Earth Planet. Sci. Lett. 481, 9–19 (2018).

[43] C. Goldfinger, C. Hans Nelson, A. E. Morey, J. E. Johnson, J. R. Patton, E. B. Karabanov, J. Gutierrez-Pastor, A. T. Eriksson, E. Gracia, G. Dunhill, R. J. Enkin, A. Dallimore, T. Vallier, Turbidite event history—Methods and implications for Holocene paleoseismicity of the Cascadia subduction zone. U.S. Geol. Surv. Prof. Pap. 1661, 170 (2012).

[44] S. Michel, J.-P. Avouac, N. Lapusta, J. Jiang, Pulse-like partial ruptures and high-frequency radiation at creeping-locked transition during megathrust earthquakes. Geophys. Res. Lett. 44, 8345–8351 (2017).

[45] P. Bak, K. Christensen, L. Danon, T. Scanlon, Unified scaling law for earthquakes. Phys. Rev. Lett. 88, 178501 (2002). 12701587 10.1103/PhysRevLett.90.109901

[46] Á. Corral, Á. González, Powerlaw size distributions in geoscience revisited. Earth Space Sci. 6, 673–697 (2019).

[47] V. Navas-Portella, Á. González, I. Serra, E. Vives, Á. Corral, Universality of power-law exponents by means of maximum-likelihood estimation. Phys. Rev. E 100, 062107 (2019). 31962489 10.1103/PhysRevE.100.062106

[48] Y. Y. Kagan, *Earthquakes*: *Models, Statistics, Testable Forecasts* (John Wiley & Sons, 2014).

[49] S. Cuven, P. Francus, S. F. Lamoureux, Estimation of grain size variability with micro x-ray fluorescence in laminated lacustrine sediments, Cape Bounty, Canadian High Arctic. J. Paleolimnol. 44, 803–817 (2010).

[50] S. Bertrand, K. A. Hughen, J. Sepulveda, S. Pantoja, Geochemistry of surface sediments from the fjords of Northern Chilean Patagonia (44–47°S): Spatial variability and implications for paleoclimate reconstructions. Geochim. Cosmochim. Acta 76, 125–146 (2012).

[51] P. J. Reimer, E. Bard, A. Bayliss, J. W. Beck, P. G. Blackwell, C. Bronk Ramsey, C. E. Buck, H. Cheng, R. L. Edwards, M. Friedrich, P. M. Grootes, T. P. Guilderson, H. Haflidason, I. Hajdas, C. Hatté, T. J. Heaton, D. L. Hoffmann, A. G. Hogg, K. A. Hughen, K. F. Kaiser, B. Kromer, S. W. Manning, M. Niu, R. W. Reimer, D. A. Richards, E. M. Scott, J. R. Southon, R. A. Staff, C. S. M. Turney, J. van der Plicht, IntCal13 and Marine13 radiocarbon age calibration curves 0–50,000 years cal BP. Radiocarbon 55, 1869–1887 (2013).

[52] M. Blaauw, J. A. Christen, Flexible paleoclimate age–depth models using an autoregressive gamma process. Bayesian Anal. 6, 457–474 (2011).

[53] C. R. P. Silver, M. A. Murphy, M. H. Taylor, J. Gosse, T. Baltz, Neotectonics of the western Nepal fault system: Implications for Himalayan strain partitioning. Tectonics 34, 2494–2513 (2015).

[54] G. Ekström, M. Nettles, A. M. Dziewoński, The global CMT project 2004–2010: Centroid-moment tensors for 13,017 earthquakes. Phys. Earth Planet. Inter. 200, 1–9 (2012).

[55] N. Lemarchand, J. R. Grasso, Interactions between earthquakes and volcano activity. Geophys. Res. Lett. 34, L24302 (2007).

[56] P. M. Kelly, C. B. Sear, Climatic impact of explosive volcanic eruptions. Nature 311, 740–743 (1984).

[57] J. P. Avouac, “Mountain building, erosion, and the seismic cycle in the Nepal Himalaya,” in *Advances in Geophysics*, R. Dmowska, Ed. (Academic Press, 2003), vol. 46, pp. 1–80.

[58] M. A. Murphy, M. H. Taylor, J. Gosse, C. R. P. Silver, D. Whipp, C. Beaumont, Limit of strain partitioning in the Himalaya marked by large earthquakes in western Nepal. Nat. Geosci. 7, 38–42 (2014).

