# Supplementary material for: Occurrence of major earthquakes is as stochastic as smaller ones
Source: Sci Adv. 2026 Feb 11;12(7):eadx7747. doi: 10.1126/sciadv.adx7747 (PMC12893319; doi:10.1126/sciadv.adx7747)
Supplement: Supplementary file 1 — Supplementary Text Figs. S1 to S7 Tables S1 to S3 References [file sciadv.adx7747_sm.pdf]

Science Advances
AAAS

Supplementary Materials for

Occurrence of major earthquakes is as stochastic as smaller ones

Zakaria Ghazoui *et al.*

Corresponding author: Zakaria Ghazoui, zahazo@bas.ac.uk

Sci. Adv. **12**, eadx7747 (2026)
DOI: 10.1126/sciadv.adx7747

This PDF file includes:

Supplementary Text
Figs. S1 to S7
Tables S1 to S3
References

Supplementary Text

Regional neotectonic setting. Western Nepal shows a more complex pattern of active faults than most other segments of the Himalaya (Fig. 1). In general, large to great earthquakes in the Himalaya are expected to nucleate on the MHT, along which the Indian plate underthrusts Tibet (e.g. (55)). In western Nepal, however, an active fault system, the Western Nepal Fault System (WNFS; Fig. 1), cuts obliquely across the Himalaya and is suggested to represent the termination of a strain-partitioned region that accommodates obliquely convergent plate motions in the western Himalaya (56, 57). Thus, the region lying between the MFT and the WNFS is a continental version of a fore-arc sliver bounded at its base by the Main Himalayan Thrust (MHT) and at its back by the WNFS (Fig. 1; (57)).

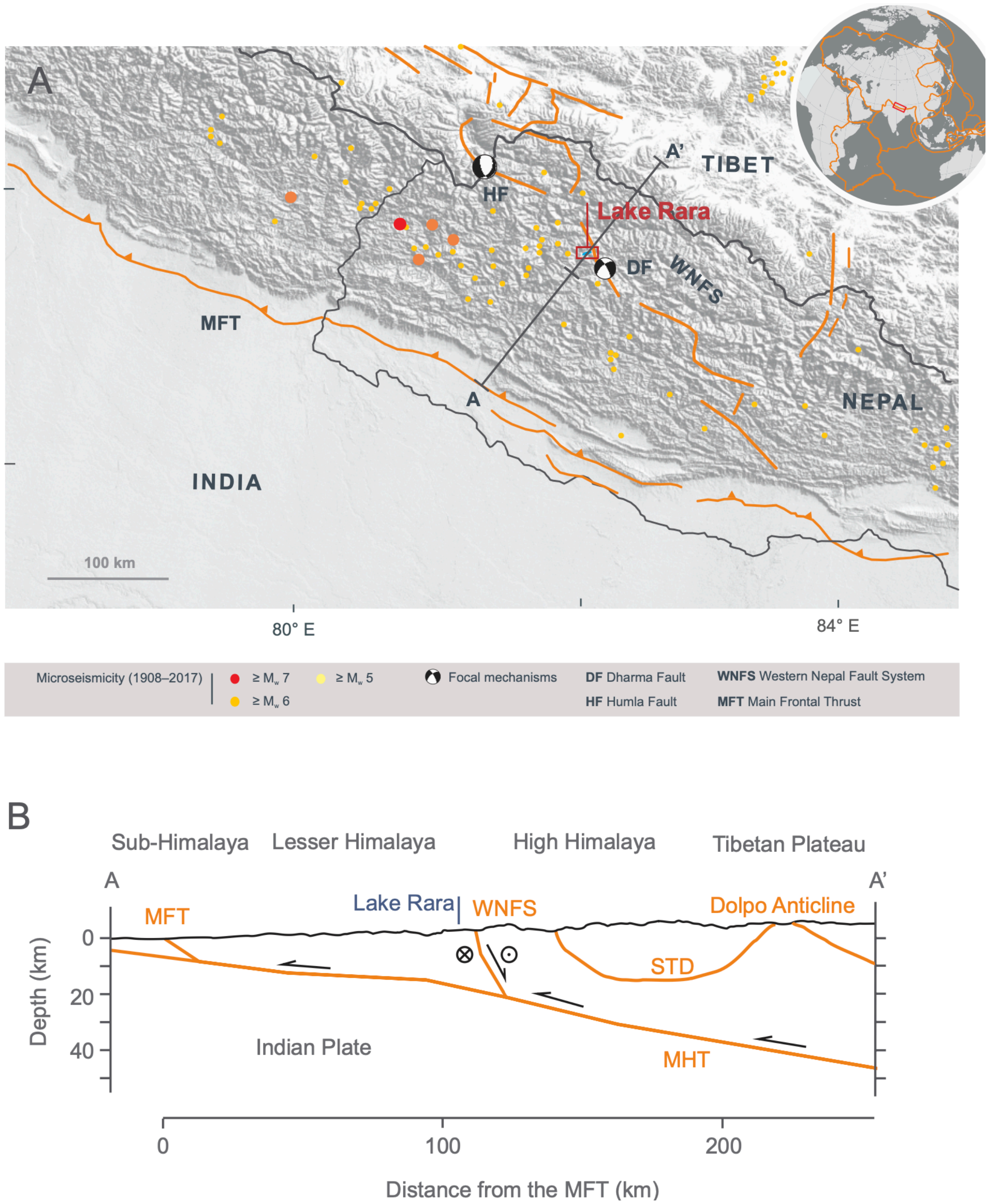

Figure S1. Map and cross-section of western Nepal showing active faults. (**A**) Neotectonic map. The red box locates Lake Rara. Regional seismicity (1908-2017, (58)) is represented by dots with colors indicating magnitude, from yellow (Mw ≥ 5) to red (Mw ≥ 7). (**B**) Schematic structural cross section across the thrust wedge highlighting the sliver geometry. Abbreviations: MHT = Main Himalayan Thrust; WNFS = Western Nepal Fault System; MFT = Main Frontal Thrust; HF = Humla fault; DF = Darma fault; STD = South Tibetan Detachment (inspired from (56, 57)).

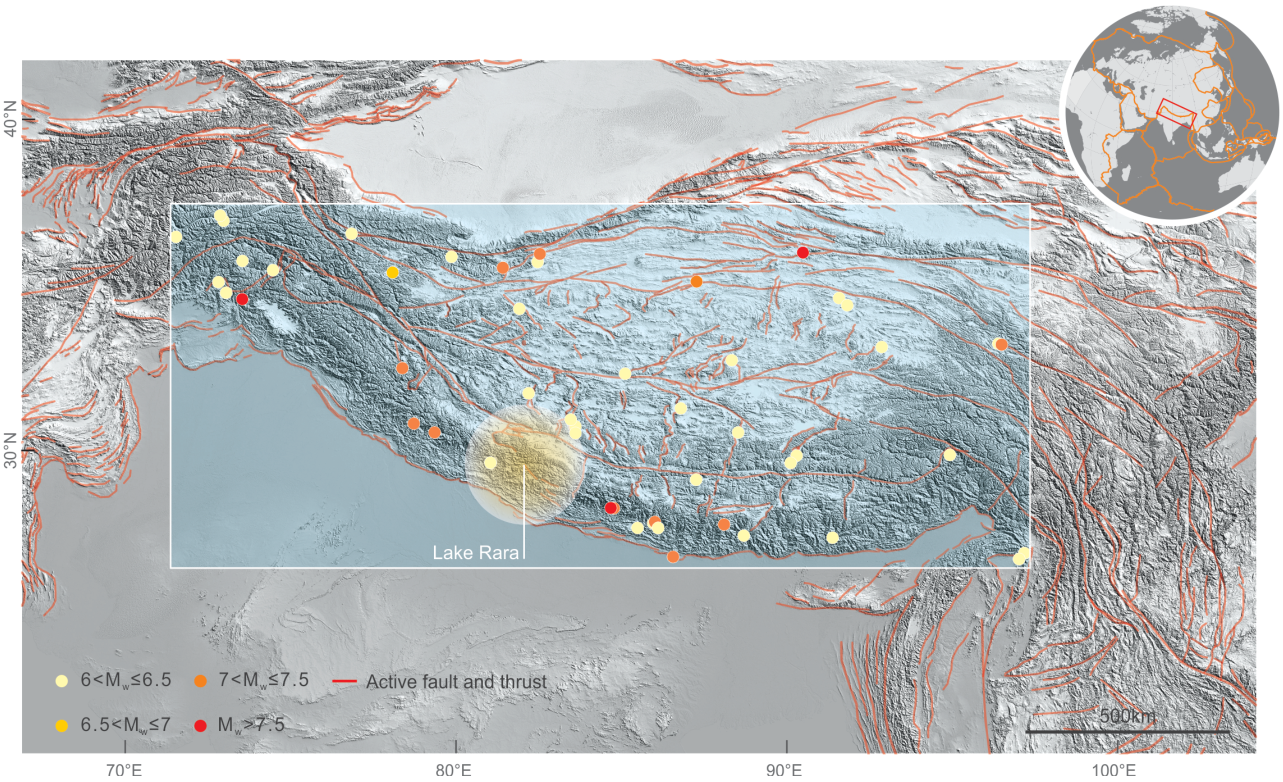

Figure S2. Map of the India-Asia collision zone with the 50largest instrumental earthquakes recorded during the period 1974-2018 (from the USGS instrumental earthquake catalogue; colored according to magnitude). The yellow circle is the area seismically resolved by Lake Rara turbidites (see Ghazoui et al. (25) for details). The blue box represents the selection area for the USGS instrumental earthquake catalogue. Orange lines are active faults.

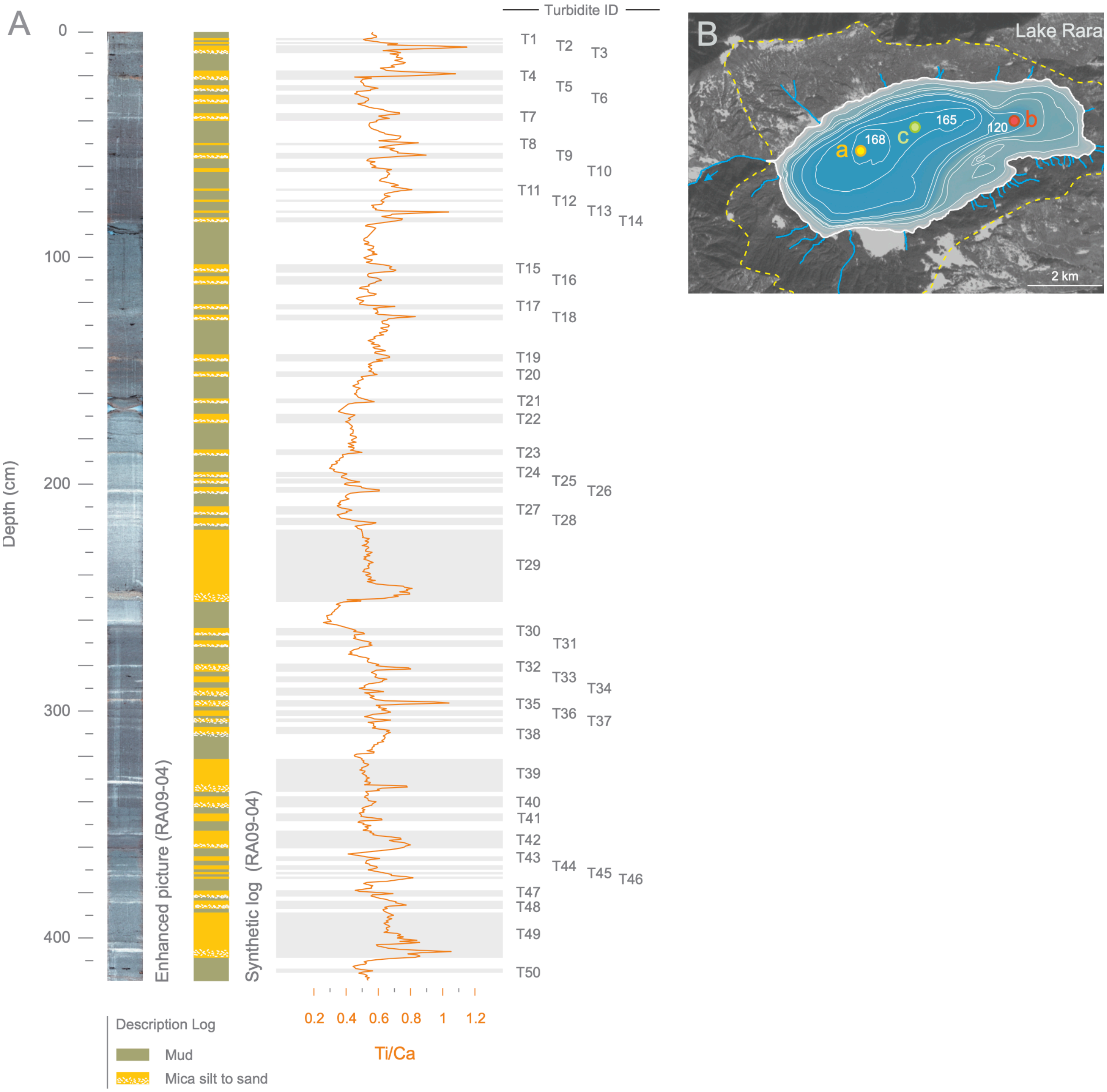

Figure S3. Sediment core RA09-04 (**A**) From left to right are shown: an enhanced core photograph, a synthetic log, XRF Ti/Ca ratio profile and the identified turbidites. (**B**) Coring site (c) of long core RA09-4 and short cores site (a and b) from previous study (25).

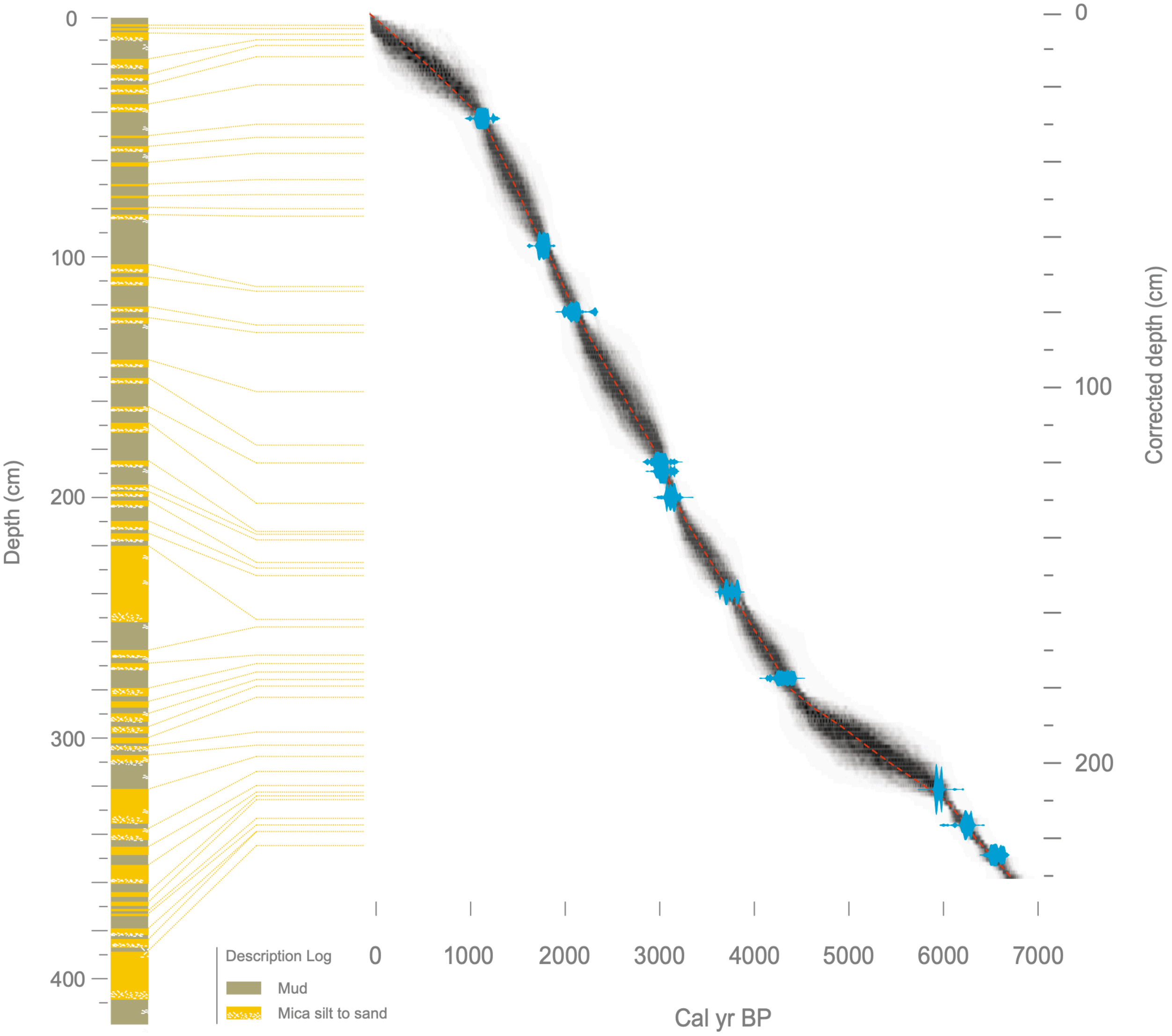

Figure S4. Bayesian age-depth model for core RA09-04. The corrected depths were calculated by removing instantaneous event deposits (turbidites) from the total depths in order to apply a continuous model in the age modeling code. Grey levels on the age-depth curves represent confidence levels. The red dashed line represents the overall best fit. Yellow dashed lines represent the projection of the turbidites on the age-depth model.

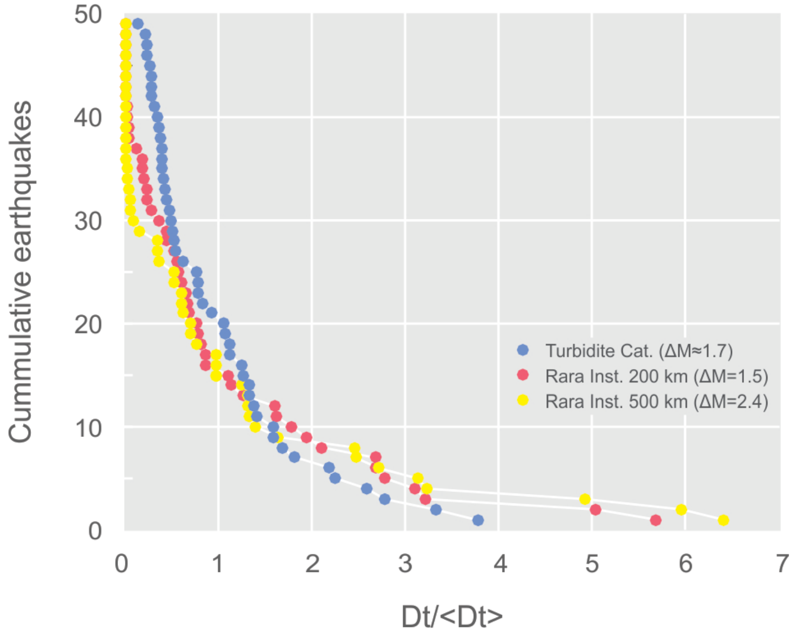

Figure S5. Collapsed inter-event time distributions from two seismic zones around Lake Rara and the ETT catalogue obtained by rescaling the inter-event times by the mean inter-event time of each distribution. (Yellow and deep-pink: USGS instrumental earthquake catalogue around Lake Rara as a radius of 500 and 200 km respectively; blue dots: ETT catalogue).

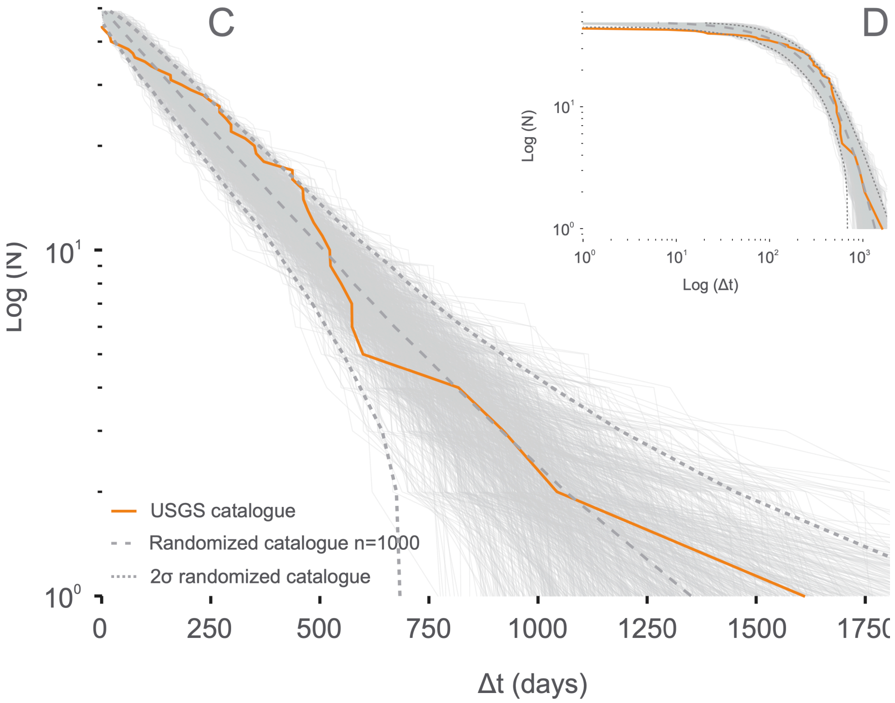

Figure S6. Plots of the cumulative distributions of earthquakes as a function of inter-event time. (**C**) Log-linear and (**D**) Log-Log plot for the instrumental catalogue. Exponential distributions reproduce the data for the instrumental catalogue (**C**, **D**; orange curve). Grey bold lines are distributions from randomized data (n=1000). The dark grey dashed curves represent the 2σ (95%) confidence level of the exponential fit. The central dashed grey curve represents the absolute reference to the Poisson model. Note the presence of expected border effects exhibiting the resolution limit for very short time intervals for both the instrumental and ETT catalogues (39).

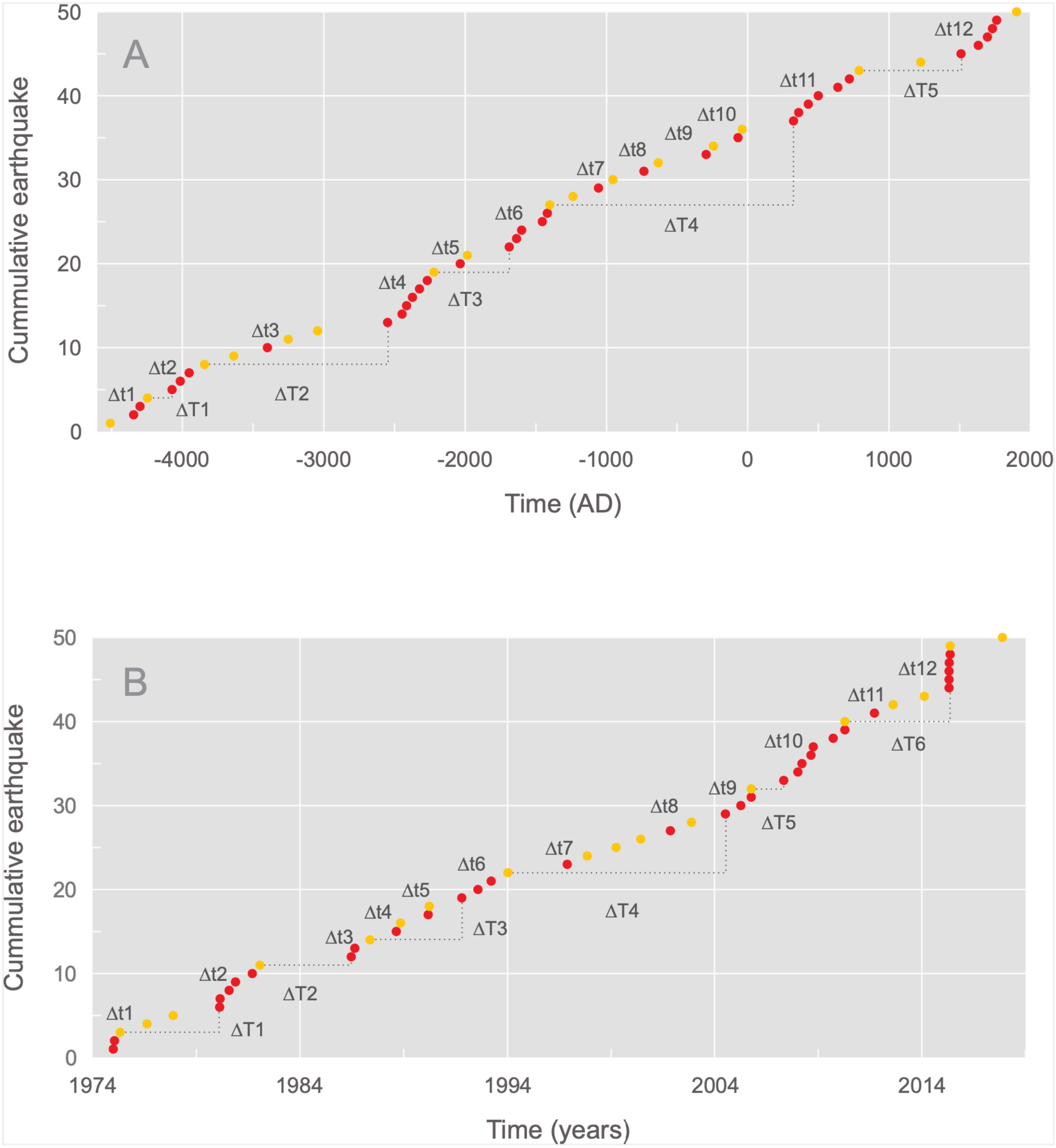

Figure S7. Visualization of cumulative number of earthquakes as a function of time for (A) the earthquake turbidite catalogue and (B) the USGS instrumental earthquake catalogue. Red dots are correlated events that are part of a seismic sequence. Yellow dots define background events. The clusters are defined (Fig. 4) by subsequent events with inter-event times (Dt_{mean}) < 148 y (**A**) or (Dt_{mean}) < 422 days (**B**). Each Δt_x and ΔT_x values correspond respectively to the cluster and the inter-sequence numbering summarized in Tables S2 and S3.

N	Weighted Mean Age (AD)	Minimum Age (AD)	Maximum Age (AD)
**1**	-4509	-4389	-4612
**2**	-4344	-4239	-4450
**3**	-4299	-4189	-4422
**4**	-4246	-4121	-4389
**5**	-4073	-3971	-4163
**6**	-4014	-3845	-4134
**7**	-3952	-3679	-4118
**8**	-3843	-3381	-4097
**9**	-3636	-3128	-3987
**10**	-3398	-2912	-3824
**11**	-3251	-2780	-3746
**12**	-3043	-2626	-3522
**13**	-2548	-2377	-2730
**14**	-2445	-2285	-2595
**15**	-2414	-2249	-2560
**16**	-2372	-2201	-2523
**17**	-2322	-2108	-2501
**18**	-2267	-2050	-2453
**19**	-2220	-2004	-2418
**20**	-2034	-1867	-2218
**21**	-1983	-1836	-2140
**22**	-1688	-1469	-1872
**23**	-1637	-1438	-1818
**24**	-1600	-1404	-1797
**25**	-1454	-1286	-1656
**26**	-1418	-1267	-1603
**27**	-1401	-1260	-1572
**28**	-1237	-1166	-1320
**29**	-1056	-921	-1156
**30**	-954	-731	-1110
**31**	-733	-465	-973

**32**	-632	-382	-893
**33**	-293	-135	-504
**34**	-241	-108	-422
**35**	-68	69	-223
**36**	-38	88	-184
**37**	324	542	168
**38**	360	579	193
**39**	428	648	244
**40**	499	710	289
**41**	638	806	462
**42**	720	871	567
**43**	787	924	654
**44**	1224	1586	933
**45**	1509	1836	1119
**46**	1632	1917	1235
**47**	1696	1941	1302
**48**	1731	1958	1349
**49**	1761	1977	1367
**50**	1902	2005	1701

Table S1. Turbidite 2σ (95% confidence level) ages of core RA09-04.

ID	n_events	Cluster onset (yr AD)	Cluster end (yr AD)	Δtx (yr)	ΔT_X (yr)
**1**	3	-4344	-4246	98	173
**2**	4	-4073	-3843	229	444
**3**	2	-3398	-3251	147	702
**4**	7	-2548	-2220	328	185
**5**	2	-2034	-1983	51	295
**6**	6	-1688	-1401	287	344
**7**	2	-1056	-954	102	221
**8**	2	-733	-632	101	338
**9**	2	-293	-241	52	173
**10**	2	-68	-38	29	363
**11**	7	324	787	462	722
**12**	6	1509	1902	392	

Table S2. Synthetic table of cluster duration for the earthquake turbidite catalogue. The clusters are defined for a minimum of 2 events separated by less than 148 years (Dt_{mean} <148 years). Δtx is the cluster duration. ΔTx is the intercluster time.

ID	n_events	Cluster onset (days)	Cluster end (days)	Δtx (days)	ΔT_X (yr)
**1**	3	0	120	120	4.8
**2**	6	1873	2583	709	4.4
**3**	3	4192	4523	331	1.2
**4**	2	4984	5060	76	1.3
**5**	2	5546	5566	19	1.5
**6**	4	6139	6953	814	2.8
**7**	2	7996	8350	353	4.0
**8**	2	9817	10189	371	1.6
**9**	4	10788	11241	453	1.5
**10**	8	11815	12890	1074	1.4
**11**	2	13413	13741	328	2.7
**12**	6	1472	14744	17	

Table S3. Synthetic table of cluster duration for the instrumental USGS catalogue. The clusters are defined for a minimum of 2 events separated by less than 422 days (Dt_{mean} <422 days). Δtx is the cluster duration. ΔTx is the intercluster time.

REFERENCES

1. L. de Arcangelis, C. Godano, J. R. Grasso, E. Lippiello, Statistical physics approach to earthquake occurrence and forecasting. *Phys. Rep.* **628**, 1–91 (2016).

2. S. C. Wu, C. A. Cornell, S. R. Winterstein, A hybrid recurrence model and its implication on seismic hazard results. *Bull. Seismol. Soc. Am.* **85**, 1–16 (1995).

3. L. R. Sykes, W. Menke, Repeat times of large earthquakes: Implications for earthquake mechanics and long-term prediction. *Bull. Seismol. Soc. Am.* **96**, 1569–1596 (2006).

4. K. Satake, B. F. Atwater, Long-term perspectives on giant earthquakes and tsunamis at subduction zones. *Annu. Rev. Earth Planet. Sci.* **35**, 349–374 (2007).

5. K. M. Scharer, G. P. Biasi, R. J. Weldon, T. E. Fumal, Quasi-periodic recurrence of large earthquakes on the southern San Andreas fault. *Geology* **38**, 555–558 (2010).

6. K. R. Berryman, U. A. Cochran, K. J. Clark, G. P. Biasi, R. M. Langridge, P. Villamor, Major earthquakes occur regularly on an isolated plate boundary fault. *Science* **336**, 1690–1693 (2012).

7. F. Corbi, F. Funiciello, M. Moroni, Y. van Dinther, P. M. Mai, L. A. Dalguer, C. Faccenna, The seismic cycle at subduction thrusts: 1. Insights from laboratory models. *J. Geophys. Res.* **118**, 1483–1501 (2013).

8. C. Goldfinger, Y. Ikeda, R. S. Yeats, J. J. Ren, Superquakes and supercycles. *Seismol. Res. Lett.* **84**, 24–32 (2013).

9. R. Herrendorfer, Y. van Dinther, T. Gerya, L. A. Dalguer, Earthquake supercycle in subduction zones controlled by the width of the seismogenic zone. *Nat. Geosci.* **8**, 471–473 (2015).

10. B. Gomez, Á. Corral, A. R. Orpin, M. J. Page, H. Pouderoux, P. Upton, Lake Tutira paleoseismic record confirms random, moderate to major and/or great Hawke's Bay (New Zealand) earthquakes. *Geology* **43**, 103–106 (2015).

11. S. J. Kenner, M. Simons, Temporal clustering of major earthquakes along individual faults due to post-seismic reloading. *Geophys. J. Int.* **160**, 179–194 (2005).

12. J. Moernaut, Time-dependent recurrence of strong earthquake shaking near plate boundaries: A lake sediment perspective. *Earth Sci. Rev.* **210**, 103348 (2020).

13. P. Kempf, J. Moernaut, Age uncertainty in recurrence analysis of Paleoseismic records. *J. Geophys. Res. Solid Earth* **126**, e2020JB021774 (2021).

14. R. Cattin, J. P. Avouac, Modeling mountain building and the seismic cycle in the Himalaya of Nepal. *J. Geophys. Res.* **105**, 13389–13407 (2000).

15. V. L. Stevens, J. P. Avouac, Interseismic coupling on the Main Himalayan Thrust. *Geophys. Res. Lett.* **42**, 5828–5837 (2015).

16. R. Bilham, V. K. Gaur, P. Molnar, Himalayan seismic hazard. *Science* **293**, 1442–1444 (2001).

17. L. Bollinger, S. N. Sapkota, P. Tapponnier, Y. Klinger, M. Rizza, J. Van der Woerd, D. R. Tiwari, R. Pandey, A. Bitri, S. Bes de Berc, Estimating the return times of great Himalayan earthquakes in eastern Nepal: Evidence from the Patu and Bardibas strands of the Main Frontal Thrust. *J. Geophys. Res.* **119**, 7123–7163 (2014).

18. T. Ader, J.-P. Avouac, J. Liu-Zeng, H. Lyon-Caen, L. Bollinger, J. Galetzka, J. Genrich, M. Thomas, K. Chanard, S. N. Sapkota, S. Rajaure, P. Shrestha, L. Ding, M. Flouzat, Convergence rate across the Nepal Himalaya and interseismic coupling on the Main Himalayan Thrust: Implications for seismic hazard. *J. Geophys. Res.* **117**, B04403 (2012).

19. Y. Kumahara, R. Jayangondaperumal, Paleoseismic evidence of a surface rupture along the northwestern Himalayan Frontal Thrust (HFT). *Geomorphology* **180**, 47–56 (2013).

20. J.-L. Mugnier, A. Gajurel, P. Huyghe, R. Jayangondaperumal, F. Jouanne, B. Upreti, Structural interpretation of the great earthquakes of the last millennium in the central Himalaya. *Earth Sci. Rev.* **127**, 30–47 (2013).

21. H. N. Srivastava, B. K. Bansal, M. Verma, Largest earthquake in Himalaya: An appraisal. *J. Geol. Soc. India* **82**, 15–22 (2013).

22. C. Schiffman, B. S. Bali, W. Szeliga, R. Bilham, Seismic slip deficit in the Kashmir Himalaya from GPS observations. *Geophys. Res. Lett.* **40**, 5642–5645 (2013).

23. S. G. Wesnousky, Y. Kumahara, D. Chamlagain, I. K. Pierce, T. Reedy, S. J. Angster, B. Giri, Large paleoearthquake timing and displacement near Damak in eastern Nepal on the Himalayan Frontal Thrust. *Geophys. Res. Lett.* **44**, 8219–8226 (2017).

24. A. Nakamura, Y. Yokoyama, H. Maemoku, H. Yagi, M. Okamura, H. Matsuoka, N. Miyake, T. Osada, H. Teramura, D. P. Adhikari, V. Dangol, Y. Miyairi, S. Obrochta, H. Matsuzaki, Late Holocene Asian monsoon variations recorded in Lake Rara sediment, western Nepal. *J. Quat. Sci.* **27**, 125–128 (2012).

25. Z. Ghazoui, S. Bertrand, K. Vanneste, Y. Yokoyama, J. Nomade, A. P. Gajurel, P. A. van der Beek, Potentially large post-1505 AD earthquakes in western Nepal revealed by a lake sediment record. *Nat. Commun.* **10**, 2258 (2019).

26. Y. Y. Kagan, P. Bird, D. D. Jackson, Earthquake patterns in diverse tectonic zones of the globe. *Pure Appl. Geophys.* **167**, 721–741 (2010).

27. M. Tahir, J. R. Grasso, Aftershock patterns of M_s>7 earthquakes in the India-Asia collision belt: Anomalous results from the Muzaffarabad earthquake sequence, Kashmir, 2005. *Bull. Seismol. Soc. Am.* **104**, 1–23 (2014).

28. I. Main, Statistical physics, seismogenesis, and seismic hazard. *Rev. Geophys.* **34**, 433–462 (1996).

29. J. G. Anderson, J. N. Brune, Methodology for using precarious rocks in Nevada to test seismic hazard models. *Bull. Seismol. Soc. Am.* **89**, 456–467 (1999).

30. A. Helmstetter, Is earthquake triggering driven by small earthquakes? *Phys. Rev. Lett.* **91**, 058501 (2003).

31. T. F. Cox, T. Lewis, Conditioned distance ratio method for analyzing spatial patterns. *Biometrika* **63**, 483–491 (1976).

32. W. Marzocchi, L. Zaccarelli, A quantitative model for the time-size distribution of eruptions. *J. Geophys. Res.* **111**, B04204 (2006).

33. Y. Y. Kagan, D. D. Jackson, Long-term earthquake clustering. *Geophys. J. Int.* **104**, 117–133 (1991).

34. J. K. Gardner, L. Knopoff, Sequence of earthquakes in southern California, with aftershocks removed, Poissonian. *Bull. Seismol. Soc. Am.* **64**, 1363–1377 (1974).

35. P. Traversa, J. R. Grasso, Brittle creep damage as the seismic signature of dyke propagations within basaltic volcanoes. *Bull. Seismol. Soc. Am.* **99**, 2035–2043 (2009).

36. M. Tahir, J. R. Grasso, D. Amorese, The largest aftershock: How strong, how far away, how delayed? *Geophys. Res. Lett.* **39**, L20308 (2012).

37. N. Ambraseys, D. Jackson, A note on early earthquakes in northern India and southern Tibet. *Curr. Sci.* **84**, 570–582 (2003).

38. R. Bilham, K. Wallace, Future Mw>8 earthquakes in the Himalaya: Implications from the 26 Dec 2004 Mw=9.0 earthquake on India's eastern plate margin. *Geol. Surv. India Spec. Publ.* **85**, 1–14 (2005).

39. S. Kumar, S. G. Wesnousky, R. Jayangondaperumal, T. Nakata, Y. Kumahara, V. Singh, Paleoseismological evidence of surface faulting along the northeastern Himalayan front, India: Timing, size, and spatial extent of great earthquakes. *J. Geophys. Res.* **115**, B12422 (2010).

40. V. L. Stevens, J. P. Avouac, Millenary M_w>9.0 earthquakes required by geodetic strain in the Himalaya. *Geophys. Res. Lett.* **43**, 1118–1123 (2016).

41. J. R. Patton, C. Goldfinger, A. E. Morey, K. Ikehara, C. Romsos, J. Stoner, Y. Djadjadihardja, Udrekh, A. Sri, E. Z. Gaffar, A. Vizcaino, A 6600 year earthquake history in the region of the 2004 Sumatra-Andaman subduction zone earthquake. *Geosphere* **11**, 2067–2129 (2015).

42. J. Moernaut, M. Van Daele, K. Fontijn, K. Heirman, P. Kempf, M. Pino, G. Valdebenito, R. Urrutia, M. Strasser, M. De Batist, Larger earthquakes recur more periodically: New insights in the megathrust earthquake cycle from lacustrine turbidite records in south-central Chile. *Earth Planet. Sci. Lett.* **481**, 9–19 (2018).

43. C. Goldfinger, C. Hans Nelson, A. E. Morey, J. E. Johnson, J. R. Patton, E. B. Karabanov, J. Gutierrez-Pastor, A. T. Eriksson, E. Gracia, G. Dunhill, R. J. Enkin, A. Dallimore, T. Vallier, Turbidite event history—Methods and implications for Holocene paleoseismicity of the Cascadia subduction zone. *U.S. Geol. Surv. Prof. Pap.* **1661**, 170 (2012).

44. S. Michel, J.-P. Avouac, N. Lapusta, J. Jiang, Pulse-like partial ruptures and high-frequency radiation at creeping-locked transition during megathrust earthquakes. *Geophys. Res. Lett.* **44**, 8345–8351 (2017).

45. P. Bak, K. Christensen, L. Danon, T. Scanlon, Unified scaling law for earthquakes. *Phys. Rev. Lett.* **88**, 178501 (2002).

46.. Corral, Á. González, Powerlaw size distributions in geoscience revisited. *Earth Space Sci.* **6**, 673–697 (2019).

47. V. Navas-Portella, Á. González, I. Serra, E. Vives, Á. Corral, Universality of power-law exponents by means of maximum-likelihood estimation. *Phys. Rev. E* **100**, 062107 (2019).

48. Y. Y. Kagan, *Earthquakes*: *Models, Statistics, Testable Forecasts* (John Wiley & Sons, 2014).

49. S. Cuven, P. Francus, S. F. Lamoureux, Estimation of grain size variability with micro x-ray fluorescence in laminated lacustrine sediments, Cape Bounty, Canadian High Arctic. *J. Paleolimnol.* **44**, 803–817 (2010).

50. S. Bertrand, K. A. Hughen, J. Sepulveda, S. Pantoja, Geochemistry of surface sediments from the fjords of Northern Chilean Patagonia (44–47°S): Spatial variability and implications for paleoclimate reconstructions. *Geochim. Cosmochim. Acta* **76**, 125–146 (2012).

51. P. J. Reimer, E. Bard, A. Bayliss, J. W. Beck, P. G. Blackwell, C. Bronk Ramsey, C. E. Buck, H. Cheng, R. L. Edwards, M. Friedrich, P. M. Grootes, T. P. Guilderson, H. Haflidason, I. Hajdas, C. Hatté, T. J. Heaton, D. L. Hoffmann, A. G. Hogg, K. A. Hughen, K. F. Kaiser, B. Kromer, S. W. Manning, M. Niu, R. W. Reimer, D. A. Richards, E. M. Scott, J. R. Southon, R. A. Staff, C. S. M. Turney, J. van der Plicht, IntCal13 and Marine13 radiocarbon age calibration curves 0–50,000 years cal BP. *Radiocarbon* **55**, 1869–1887 (2013).

52. M. Blaauw, J. A. Christen, Flexible paleoclimate age–depth models using an autoregressive gamma process. *Bayesian Anal.* **6**, 457–474 (2011).

53. C. R. P. Silver, M. A. Murphy, M. H. Taylor, J. Gosse, T. Baltz, Neotectonics of the western Nepal fault system: Implications for Himalayan strain partitioning. *Tectonics* **34**, 2494–2513 (2015).

54. G. Ekström, M. Nettles, A. M. Dziewoński, The global CMT project 2004–2010: Centroid-moment tensors for 13,017 earthquakes. *Phys. Earth Planet. Inter.* **200**, 1–9 (2012).

55. N. Lemarchand, J. R. Grasso, Interactions between earthquakes and volcano activity. *Geophys. Res. Lett.* **34**, L24302 (2007).

56. P. M. Kelly, C. B. Sear, Climatic impact of explosive volcanic eruptions. *Nature* **311**, 740–743 (1984).

57. J. P. Avouac, "Mountain building, erosion, and the seismic cycle in the Nepal Himalaya," in *Advances in Geophysics*, R. Dmowska, Ed. (Academic Press, 2003), vol. 46, pp. 1–80.

58. M. A. Murphy, M. H. Taylor, J. Gosse, C. R. P. Silver, D. Whipp, C. Beaumont, Limit of strain partitioning in the Himalaya marked by large earthquakes in western Nepal. *Nat. Geosci.* **7**, 38–42 (2014).
